# Evaluation of the Effectiveness of Standardized Patient Simulation as a Teaching Method in Psychiatric and Mental Health Nursing

**DOI:** 10.3390/nursrep14020107

**Published:** 2024-06-04

**Authors:** Eman Dawood, Sitah S. Alshutwi, Shahad Alshareif, Hanaa Abo Shereda

**Affiliations:** 1 College of Nursing, King Saud Bin Abdulaziz University for Health Sciences, Riyadh 11481, Saudi Arabia; dawoode@ksau-hs.edu.sa (E.D.); shareifs@ksau-hs.edu.sa (S.A.); aboshereidah@ksau-hs.edu.sa (H.A.S.); 2 King Abdullah International Medical Research Center, Riyadh 14611, Saudi Arabia; 3 Department of Psychiatric and Mental Health Nursing, College of Nursing, Menoufia University, Shibin El Kom 32511, Egypt

**Keywords:** standardized patient, simulation, psychiatric and mental health nursing, self-confidence, satisfaction, nursing education, nursing practice, Saudi Arabia

## Abstract

*Background:* The use of standardized patient simulation in psychiatric nursing education addresses the unique challenges presented by mental healthcare settings. Students’ attitudes toward clinical simulation remain predominantly favorable, with many expressing enthusiasm for the opportunities it provides in terms of embracing challenges, enhancing realism, and promoting critical thinking through problem solving, decision-making, and adaptability. *Methods:* This quantitative study used a cross-sectional, descriptive, correlation design to investigate the effectiveness of standardized patient simulation as a teaching method in the Psychiatric and Mental Health nursing course in a university setting. A total of 84 nursing students were recruited for the convenience sample. Data were collected using a three-part questionnaire survey which included the following: a demographic data sheet, the Student Satisfaction and Self-confidence in Learning Scale, and a narrative open-ended question asking the participants to write the advantages and disadvantages of their simulation experience. Data were analyzed using the statistical software JMP pro17. *Results:* The total satisfaction with learning subscale score ranged between 5 and 25 with a mean score of 19.36 ± 6.32. The total self-confidence subscale score ranged between 8 and 40 with a mean score of 30.87 ± 9.1. Pearson’s correlation coefficient r revealed a statistically significant positive relationship between the participants’ satisfaction with the learning experience and their self-confidence (t = 0.923, *p* < 0.0001). Approximately 91.7% of the students recommended using simulation. The results confirmed the students’ recommendations of simulation use in teaching psychiatric and mental health courses; furthermore, the results showed a statistically significant positive correlation with the total SSLS (*p* = 0.01) and satisfaction with learning subscale (0.003). Participants reported that authentic, practical, comfortable, and safe learning environments contributed to an enriched learning experience. Additionally, factors such as timesaving, access to information, cost-effectiveness, standardized teaching, varied exposure, skill development, and immediate feedback also enhanced the learning experience through patient simulation in psychiatric and mental health nursing. *Conclusion:* Simulations can contribute efficiently and positively to psychiatric and mental health nursing education in a manner that optimizes the learning experience while ensuring the consistency of student learning in a safe learning environment.

## 1. Introduction

Psychiatric nursing education is a critical component of the nursing curricula, where students are to be equipped with the essential qualities, knowledge, and skills to deliver compassionate and efficient care to patients with mental health disorders. The coursework encompasses a curriculum that prioritizes practical experience, focusing on theoretical understanding and psychomotor abilities. In skill-based education, where hands-on learning is paramount, effectively blending theoretical understanding with practical application is crucial. In this context, simulation emerges as a progressive instructional approach, simultaneously engaging multiple senses in learners to achieve the intended learning outcomes [1].

Simulation has progressively gained traction as a crucial teaching strategy in nursing education [2]. In general, it involves replicating tasks, connections, occurrences, phenomena, conduct, and cognitive processes that are present in real life [3,4]. This pedagogical method aims to mimic real-life scenarios [5]. Standardized patients are expertly trained people who mirror actual patients, offering a highly authentic experience that can greatly help nursing students throughout their learning experience [6]. Current research offers enough evidence to validate SP simulations as an effective method to improve students’ clinical abilities. Simulation, with its safe and professionally supervised environment for clinical competencies training, is essential in preparing nursing students for real-world clinical scenarios. In psychiatric nursing, SPs allow students to improve their knowledge and competencies in addition to learning from errors by engaging with actual people through interactions and obtaining tangible feedback on how they perform. Moreover, SP simulation enhances students’ preparedness for clinical placement with actual patients by decreasing their levels of anxiety and stress by increasing their knowledge, confidence, and interpersonal communication skills [1,7,8,9].

The use of standardized patient simulation in psychiatric nursing education can address the unique challenges presented by mental healthcare settings. Following every simulation-based learning experience, a professionally prepared skilled facilitator leads an arranged debriefing session. During the debriefing, the facilitator’s target is to foster a supportive environment where participants can discuss their thoughts, clinical skills, actions taken (or not taken) with rationale, and the gaps in their knowledge and abilities while reflecting on their simulation experience and how it relates to their future clinical practice [10]. Recent evidence-based practices revealed that simulation training in psychiatry could be enhanced by tailoring it to encompass a broad spectrum of mental disorders, acknowledging the unique social and cultural factors, and considering different life stages to embrace diversity and specificity [11].

Students’ attitudes toward clinical simulation remain predominantly favorable, with many expressing enthusiasm for the opportunities it provides in terms of embracing challenges, enhancing realism, and promoting critical thinking [12]. Consequently, students’ confidence in clinical practice will grow, their anxiety levels will decrease, and they will become highly motivated to engage in practical learning [1,13].

The Psychiatric and Mental Health nursing course is unique because dealing with patients experiencing mental health problems requires unique skills. Compared with traditional teaching methods, simulation offers the opportunity to develop and enhance verbal and nonverbal communication skills, interviewing, assessment, and intervention skills and boost critical thinking [14].

Only one large governmental specialized psychiatric mental health hospital affiliated with the Ministry of Health in Riyadh, Saudi Arabia, provides clinical training for all healthcare professional students and trainees. Unfortunately, this situation places a huge overload on the patients receiving care at this hospital, as well as the medical and administrative staff managing all these students, along with providing efficient care to patients, considering their rights, and protecting their confidentiality. Another crucial aspect is the unique nature of psychiatric patients and the complex requirements of this particular population; exposure to many trainees most of the time would hinder the treatment plans and schedules of patient activities. In addition, the stigma of psychiatric illness still has a major effect on the Saudi community’s perception of mental illness. Thus, the hospital has very strict policies and regulations in allowing trainees to deal with psychiatric patients with different psychiatric problems, which in turn exerts a huge amount of pressure on the educational institution to consider some innovative alternatives to overcome these obstacles. Another important point is the increased number of nursing students every year. Therefore, standardized patient simulation must be utilized in teaching psychiatric nursing skills and competencies to ensure that students can receive the best experience necessary for developing their profession and be well-prepared for their actual clinical practice and prospective future careers as psychiatric and mental health nurses.

An extensive literature review revealed the scarcity of the available literature and evidence of the effectiveness of simulation in psychiatric nursing education within the Saudi community considering the unique cultural criteria. Therefore, this study aimed to evaluate the effectiveness of standardized patient (SP) simulation in teaching the undergraduate Psychiatric and Mental Health nursing course as a part of the Bachelor’s degree of nursing at the College of Nursing (CON-R), affiliated with King Saud Bin Abdulaziz University for Health Sciences in the central region of Saudi Arabia.

### Aim of the Study

This study aimed to investigate the effectiveness of standardized patient simulation as a teaching method in the Psychiatric and Mental Health nursing course in a university setting in Riyadh, Kingdom of Saudi Arabia.

## 2. Materials and Methods

A descriptive correlation cross-sectional design was utilized for this research. This study adhered to the Strengthening the Reporting of Observational Studies in Epidemiology (STROBE) guidelines. This study was conducted at the College of Nursing affiliated to the King Saud bin Abdelaziz University for Health Sciences (KSAU-HS) in the central region of Saudi Arabia. The inclusion criteria were as follows: undergraduate student nurses who enrolled in the Psychiatric and Mental Health nursing course and who consented to voluntarily participate in this study. The sample size was determined as nursing students enrolled in the Psychiatric and Mental Health nursing course (96 students) with a confidence level of 95% and a margin of error of 5% using the sample size calculator (Roasoft) software [15]. The response distribution was 50%, and the calculated sample size was 77 nursing students. The actual number of students who met the inclusion criteria and consented to participate was 84. A convenience sampling technique was utilized. Considering the inclusion criteria, 84 student nurses who enrolled in the Psychiatric and Mental Health nursing course were recruited and were exposed to standardized patient simulation as a teaching strategy in the College of Nursing affiliated to KSAU-HS in the central region of the Kingdom of Saudi Arabia. Standardized patients were recruited from the university simulation center and trained by the course team; all of the scripts were prepared and extensively reviewed by experts in the area of psychiatric nursing. Students were exposed to five simulation sessions, followed by six hospital-based clinical training sessions at the mental health hospital.

Data were collected using a three-part questionnaire survey. Part one was the demographic data sheet developed by the researchers. It included age, GPA, past experience with simulation, and past exposure to people with mental health problems. Part two was the Student Satisfaction and Self-confidence in Learning Scale developed and validated by the National League of Nursing in 2006 [16]. The scale was utilized to assess the students’ satisfaction with the simulation experience and their self-confidence levels after learning. It comprises 13 questions with two subscales assessing satisfaction (five questions) and self-confidence (eight questions). This 5-point Likert scale has a response option ranging from 5 (strongly agree) to 1 (strongly disagree). No cut-off score was set, so the mean was utilized to represent the levels of students’ satisfaction and self-confidence. This scale has been used widely by different researchers globally and has been reported to have good reliability, confirmed by Cronbach’s alpha coefficient which ranged between 0.90 and 0.94 for the satisfaction subscale and 0.87 and 0.91 for the self-confidence subscale [17,18]. The third part included open-ended questions asking the participants to describe their experience with the simulated patient in their own words and to list the advantages and the disadvantages of using standardized patient simulation in teaching psychiatric and mental health nursing from their own perspectives. The questionnaire was in English, which is the language of study in the institution.

Review and approval were obtained from the research unit at the College of Nursing, KSAU-HS. Ethical approval was granted via the Institutional Review Board Committee (IRB) at KAIMRC (NRC23R/700/11). All the participants were instructed about voluntary participation in this study and were assured that they had the right to withdraw from the study at any time without any penalty or effect on their course grades by any means. Data were collected after the purpose and nature of the study were explained and informed consent was secured. Neither any known harm resulting from participation in the study nor any gained entitlement was present. Voluntary participation, confidentiality, and anonymity were ensured. Each participant was given a hard copy of the Student Satisfaction and Self-confidence in Learning Scale along with the demographic and academic-related data sections to complete and return anonymously to the researchers at the end of the debriefing session following the last clinical simulation session at CON-R. The narrative part of the questionnaire was distributed and collected from the participants after the last clinical practice visit. The questionnaire was anonymous and no identifying data that might reveal the identity of the participants were asked. All collected data were kept confidential and used only for this study.

The statistical software JMP Pro17 was utilized to analyze quantitative data. Once the completed surveys were received from the participants, the data were immediately coded, entered, and cleaned into JMP. A descriptive statistics method was adopted to describe the sample characteristics and evaluate whether the results were normally distributed or not. Appropriate inferential statistical tests were used to determine the strength and direction of the relationships among study variables. The significance level was chosen as (*p* < 0.05).

## 3. Results

### 3.1. Sociodemographic Characteristics of the Study Sample

Data were collected from student nurses who enrolled in the Psychiatric and Mental Health nursing course at the College of Nursing, KSAU-HS, Riyadh, and who consented to voluntarily participate in this study. This work aimed to investigate the effectiveness of standardized patient simulation as a teaching method in the Psychiatric and Mental Health nursing course in a university setting in Riyadh, Kingdom of Saudi Arabia. Eighty-four participants who completed the self-reported questionnaire were included in this study. Table 1 shows the sociodemographic criteria of the study sample. The participants’ ages ranged between 20 and 23 years, with a mean age of 21.21 ± 0.79 years. The students’ GPA scores ranged between 2.33 and 4.95 points, with a mean of 4.09 ± 0.44. More than three-quarters of the participants (76.2%) had a past experience with simulation in previous clinical nursing courses, and more than half of them (65.5%) had received some simulation training. Furthermore, 56% of the students have had a past experience with a person experiencing a mental health problem.

### 3.2. Level of Comfort with Interviewing and Managing a Patient with a Mental Health Problem in Real Clinical Settings

The participants were asked to rate their comfort level to explore their level of comfort with interviewing and managing a patient with a mental health problem in a real clinical setting. The results revealed that 42.9% of participants reported that they were extremely or very comfortable with interviewing and managing a patient with a mental health problem in a real clinical setting. More than half of the participants (57.1%) were either slightly or not at all comfortable with either interviewing or managing a patient with a mental health problem in a real clinical setting (Table 2).

### 3.3. Recommendation of Using a Simulated Patient as a Teaching Method in Teaching Psychiatric and Mental Health Nursing

Table 3 presents the students’ recommendations for patient simulation as a teaching method for psychiatric and mental health nursing after their real experience. More than three-quarters (91.7%) of the students recommended using a simulation, and only seven students did not recommend it.

### 3.4. Frequency Distribution of Students’ Satisfaction with Learning Scale Items

The total SSLS score ranged between 13 and 65, with a mean score of 50.27 ± 15.13. The participants were asked to indicate how much they agreed or disagreed with each scale statement associated with their point of view toward satisfaction with the simulation learning experience to assess their satisfaction level with psychiatric nursing simulation teaching. Table 4 illustrates the participants’ responses. The total satisfaction with learning subscale score ranged between 5 and 25, with a mean score of 19.36 ± 6.32.

### 3.5. Frequency Distribution of Students’ Level of Self-Confidence in Learning

Table 5 illustrates the level of confidence in learning via the simulation teaching experience of the participants. They were asked to indicate how much they agreed or disagreed with each scale statement associated with their point of view toward self-confidence in a learning experience with a simulation. The total self-confidence subscale score of the study participants ranged between 8 and 40, with a mean self-confidence subscale score of 30.8 ± 79.1.

### 3.6. Correlation between Participants’ Satisfaction with Learning and Self-Confidence

Pearson’s correlation coefficient r revealed a statistically significant positive relationship between the participants’ satisfaction with the learning experience and their self-confidence (t = 0.923, *p* < 0.0001, with a confidence interval from approximately 0.88 to approximately 0.95).

### 3.7. Relationship between Satisfaction with Learning and Past Experience with Simulation, Simulation Training, and Past Experience with a Person with a Mental Health Problem among Study Participants

As shown in Table 6, ANOVA revealed no difference between groups with regard to their satisfaction with the learning scale score, past experience with simulation, simulation training, and past experience with a person with a mental health problem (*p* = 0.224, 0.258, 0.090, respectively).

### 3.8. Relationship between Self-Confidence in Learning Scale Score and Past Experience with Simulation, Simulation Training, and Past Experience with a Person with a Mental Health Problem among Study Participants

As presented in Table 7, ANOVA revealed no difference between groups with regard to their self-confidence in learning and past experience with simulation, simulation training, and past experience with a person with a mental health problem (*p* = 0.478, 0.480, 0.129, respectively).

### 3.9. Relationship between Total SSLS Scores and Past Experience with Simulation, Simulation Training, and Past Experience with a Person with a Mental Health Problem among Study Participants

As presented in Table 8, one-way ANOVA revealed no difference between groups with regard to total Students’ Satisfaction and Self-confidence in Learning Scale scores and past experiences with simulation, simulation training, and past experiences with people with mental health problems (*p* = 0.350, 0.369, 0.105, respectively).

### 3.10. Correlation between Satisfaction, Self-Confidence, Total SSLS and Students GPA

As presented in Table 9, no relationship was detected between the students’ grade point average and either the subcategories or the total SSLS scores (*p* = 0.250, 0.329, 0.286, respectively).

### 3.11. Correlation between Satisfaction, Self-Confidence, Total SSLS and Students’ Recommendation to Use Simulation in Psychiatric and Mental Health Education

As illustrated in Table 10, data analysis confirmed a statistically significant positive correlation between the students’ recommendations of simulation use in teaching psychiatric and mental health courses with the total SSLS (*p* = 0.01) and their satisfaction in learning subscale (0.003). Meanwhile, no significant relation was detected between their self-confidence in learning subscale (0.06) and their high level of recommendation of the simulation and high score in the total SSLS and its two subscales.

### 3.12. Responses to Open-Ended Questions Related to the Advantages and Disadvantages of Using Simulated Patients in Teaching a Psychiatric Nursing Course

For enrichment, the researchers added open-ended questions to allow the participants to express and verbalize their personal opinions related to the advantages and disadvantages of using simulated patients in teaching psychiatric nursing courses. Their feedback was as follows:

*Advantages:* In total, 69 (82.14%) of the students who participated in the study found the simulation experience useful and helpful in understanding and learning how to ask questions, initiate interactions, and interview patients. Additionally, 63.1% (53) students mentioned it was useful in building and developing knowledge and obtaining important information and gave a feeling of a real patient assessment as stated by students’ quotes, “I learned how to ask questions to the patient knowing that the patient was able to answering since it is a Simulated PT. I liked the experience, and it was helpful for me and provided all the information I needed”. A couple of participants valued this experience as it made gaining knowledge and learning easier than with real patients due to the time and communication it afforded them. In addition, 52.4% of the students highlighted the effectiveness of immediate feedback and the safe learning environment. Moreover, 77.4% of students said “My instructor explained to us how to communicate with simulated patients with enough time” and “simulation experience helped me with Mental Status Examination and Nursing Care Plan because we had enough time to collect data”.

*Safe learning environment and access information:* This approach creates a safe place where students can communicate, interact, and gather a large amount of information without interruption, fear of stress, or making mistakes. In total, 71 students said the process “helps exploring the psychiatric patients’ symptoms in safe environments”, “Simulated patients were cooperative and answered all questions, aiming in information collection”, and “less distracting environment, good communication”. The participants stated that this learning technique offers considerable exposure to mental illness cases through simulation and noted “now, I can expect how real patients would look like from the simulation sessions and I am ready to manage different patients’ symptoms”. Additionally, 39 students stated that patient simulation gives great opportunities for practicing and exploring symptoms in a safe and controlled environment. Some students stated the process “provides a variety of mental illness and help us to learn” and “Using simulated patients as a teaching method offers advantages such as realistic practice, immediate feedback, and psychiatric nursing skill development”. The advantages highlighted were authenticity, practicality, comfort, safe learning environments, timesaving, access to information, cost-effectiveness, standardized teaching, varied exposure, skill development, and immediate feedback. All of these factors contributed to an enriched learning experience through patient simulation in psychiatric and mental health nursing.

The disadvantages were as follows: in total, 11 students reported some drawbacks of their simulation experience as unclear or inconsistent responses from the simulated patient, e.g., “Some simulated patients frequently answered with ‘I don’t know’ or responded unclearly, giving ambiguous answer that hindered my assessments and made me confused while gathering of necessary information”.

*Group dynamics:* The learning experiences in groups were distracting to some students and made it challenging for them to concentrate and collect the data; “it was in groups, so it was distracting”, as reported by four students.

*Simulated patient’s inability to adhere to the scenario:* “Simulated patients occasionally diverged from the main scenario, which made accurate assessment hard and challenging to conduct properly”, and “some of simulated PT did not follow the written scenario” as reported by six participants.

Overall, the experiences were mixed and diverse. The majority of students found simulated patient interactions immensely beneficial in a safe learning environment and a realistic portrayal of patients. Others faced challenges related to communication, time constraints, and inconsistencies in patient responses and desired additional exposure to real patient scenarios for a complete clinical learning experience.

## 4. Discussion

Teaching the Psychiatric and Mental Health nursing course to undergraduate students is challenging as the majority of nursing students have a negative attitude, anxiety, and fear of being in direct contact with patients experiencing mental health problems; this situation hinders the students’ learning [19]. Simulation-based learning has become an integral part of nursing education worldwide, especially in preparing students to deal with real-life patient scenarios. Therefore, this study aimed to explore the relationship between nursing students’ satisfaction with simulation learning and their self-confidence in dealing with real patients experiencing mental health problems following their experience. This critical point was not discussed clearly in previous simulation-related studies.

The experience afforded the students the opportunity to gain exposure to real-life events and some clinical situations they may not encounter in their actual practice setting. The learning experience in mental health settings is opportunistic because of the expected variance in patients’ symptoms due to the nature of mental illnesses.

The participants’ ages ranged from 20 to 23 years, and their GPA mean score was 4.09 ± 0.44. These findings are consistent with those of Alharbi et al. and Khalil et al. [17,20] who reported that the range of the participants’ ages in their studies was 20–22 years with a GPA average of 3.79.

Regarding the satisfaction level of psychiatric nursing students with standardized patient simulation, this study found that the total satisfaction with the learning subscale of the study participants ranged between 5 and 25, with a mean score of 19.36 ± 6.32. This finding indicates that participating students were highly satisfied with the use of simulated patients in learning psychiatric nursing clinical competencies. These results are congruent with recent studies by Amsalem et al., Ma et al., Ozkara San et al., and Presno et al. [21,22,23,24], indicating that standardized patient simulation can effectively improve undergraduate nursing students’ learning satisfaction in mental health nursing and help to prepare them for their real clinical placements. In addition, Johnson et al. and Goh et al. [25,26] found that using simulated patients in undergraduate mental health nursing education significantly increased students’ satisfaction before being posted to mental health settings for their clinical attachment.

Regarding psychiatric nursing students’ confidence levels in dealing with real patients with mental health problems following their learning experience with standardized patient simulations; in terms of confidence levels, the participating students showed a high level of self-confidence in dealing with real psychiatric patients after experiencing simulated patients. Several studies found that simulated patient-based training endows students with high levels of self-confidence by improving their clinical competencies and communication skills in psychiatric settings and enhancing their abilities to carry out clinical tasks and abilities to work in teams. Moreover, the participating students felt confident and prepared for real-world encounters with mental health patients [21,24,26,27,28,29,30]. Meanwhile, Johnson et al. [25] reported that using standardized patient simulation for first-semester nursing students was not effective in improving their self-confidence. This finding can be rationalized as follows: the subjects in this study were beginning-level nursing students who generally lack confidence in their first contact with patients, whether real or simulated.

Correlation between satisfaction and self-confidence: The integration of standardized patient simulations in teaching clinical psychiatric nursing competencies has been proven to be a promoting experience that increases the participating students’ satisfaction. It consequently enhances their self-confidence level, as evidenced by this study, indicating a positive correlation between the students’ satisfaction and confidence. Furthermore, the participating students enjoyed the standardized patient simulation as they became highly confident in developing the required knowledge and clinical skills from the nursing simulation [31,32,33].

The qualitative section of the study focused on students’ opinions about the simulations and the advantages they gained after their experience. Many students found the simulation experience to be highly useful. It effectively bridged the gap between the theoretical knowledge they acquired and its practical application in clinical situations. Several students highlighted the significant benefit of the simulation in helping them initiate interactions and interview real patients in mental health settings. Many students perceived such tasks as distressing, especially during their initial contact with patients experiencing different mental health problems.

The students highlighted other factors that emphasized the usefulness of the simulation training, which are as follows: The simulation felt realistic and presented a variety of psychopathology and psychiatric disorders, which in turn increased their satisfaction and self-confidence in dealing with real psychiatric patients. In addition, it provided a safe environment for students to cultivate and enhance their skills without being scared of dealing with mentally disturbed patients, at least in the initial clinical training phase. Furthermore, the availability of time to finish the interview with the patient and to receive feedback from the clinical instructor at the end of every simulation scenario allowed the students to learn from their mistakes.

Although most students’ comments indicated that the use of standardized patient simulation was beneficial for the majority of students, it could be considered incongruent as expressed: “How can we provide a learning environment that becomes both safe and at the same time reflects the unpredictability of clinical practice especially with psychiatric and mentally ill patients?” Therefore, a balance should be achieved and was actually considered while creating and implementing the simulation experience between the fictional cues, “which are those artifacts, actions, perceptions, structures and situations that emphasize the artificial nature of the experience,” and reality cues to make the experience more similar to a real-life clinical psychiatric setting [34].

Most of the students reported that the standardized patient simulation was a very engaging experience that increased their abilities to deal with real psychiatric patients. Godzik et al. [29] also concluded that students established a high level of engagement with the standardized patients, leading to a deep level of understanding and learning that was reflected in their opinions. On the contrary, Berragan [35] stated that simulation limits students’ engagement with the social and cultural words of nursing. In this context, integrating patient simulation with real-life clinical training, starting with simulation and ending with real-life training, could optimize the benefits derived from both simulation and real-life training.

The participants revealed some disadvantages of the standardized patient simulation experience. A few students found that the inability of the standardized patient to adhere to the written scenario sometimes caused them to become confused and unable to complete their clinical competency spontaneously. This comment disclosed a critical point in the design and preparation of standardized patient simulation; the preparation of well-structured scenarios and instructions for simulated patients requires careful monitoring and continual adjustments to incorporate the learners’ needs. In addition, the creation, validation, training, and rehearsal of clinical scenarios is time-consuming and requires constant work and modifications. The time constraints may prompt the trainer to prepare a simple, short, and directive scenario [11].

## 5. Conclusions

Psychiatric mental health nursing simulations can be useful learning tools for engaging in a variety of mental health nursing scenarios in a safe, simulated learning environment. In addition to the efforts devoted to the protection of psychiatric patients’ rights and providing high-quality psychiatric patient care, the growing number of nursing students enrolling in the College of Nursing led to a decrease in the nursing students’ direct contact time with psychiatric patients. Therefore, SP simulation is being used to help resolve all these challenges and provide high-quality psychiatric nursing education, especially because it works for individuals who have difficulty forming interpersonal relationships or communicating. Although many studies have been conducted on the use of simulation in nursing, the current work is unique because it focuses on the nursing students’ self-confidence and experience in dealing with real psychiatric patients after the SP simulation. The results revealed that nursing students’ satisfaction and self-confidence were significantly improved after the SP simulation. Moreover, the students’ self-confidence in dealing with real psychiatric patients increased after attending the SP simulation. In addition, the nursing students found the SP simulation to be an effective method that helps them bridge the gap between theory and clinical practice in a safe, controlled environment that mimics real-life clinical training.

## 6. Limitation of the Study

Although this study provided new evidence for the use of SP simulations in mental health nursing education in Saudi Arabia as an example of an Arab country with its unique culture and traditions, there are some limitations, which include the following: the use of convenience sampling in which the students were not randomly selected in addition to the small sample size from only one setting (CON-R) can decrease the generalizability of the results. The use of a self-rating scale can produce response bias. Moreover, adopting the cross-sectional research design may be a limiting factor. Further, although the SPs have undergone rigorous training on the selected competencies of psychiatric and mental health nursing course still due to the unique nature of psychiatric psychopathology presentation and their limited knowledge of psychiatric and mental disorders, the consistency and realism of the simulation may have been affected in a few occasions during the simulation sessions.

## 7. Recommendations

Based on the findings of this study, SP simulation must be incorporated in teaching Psychiatric and Mental Health nursing courses. The focus of these simulations should be on advanced communication, mental status examination skills, and emergencies that can be further reinforced when students engage in their real clinical placements. Given the importance of the sequence of SP simulation, commencing with SP simulation prior to the actual real clinical experience is crucial in decreasing the stress, anxiety, and fear of dealing with real patients with mental health problems. Subsequent to the SP simulation is real clinical placement to provide the unpredictable experience of real clinical training and determining how to deal with real patients experiencing mental health problems. Additional studies are necessary to understand whether nursing students who worked with simulated patients prior to real clinical settings perform better than their counterparts. Designating enough funding to universities and colleges to construct standardized patient simulation laboratories would allow instructors to integrate this learning strategy into their curriculum, thus producing confident, well-prepared, and knowledgeable nursing students who can carry out the responsibilities of future psychiatric nurses. Future multisite research studies with large samples utilizing diverse research methodologies and including a wide variety of mental health nursing scenarios that address different clinical competencies and skills are highly recommended.

## Figures and Tables

**Table 1:** Sociodemographic characteristics of the study sample (*n* = 84).

Variable	Frequency (N)	Percent (%)
**Age** Mean 21.21 SD ± 0.79		
**Grade Point Average (GPA)** Mean 4.09 SD ± 0.44		
**Do you have any past experience with simulation?** Yes No	64 20	76.2 23.8
**Have you ever received any simulation training?** Yes No	55 29	65.5 34.5
**Do you have any past experience with a person experiencing a mental health problem?** Yes No	47 37	56 44

**Table 2:** Level of comfort with interviewing and managing a patient with a mental health problem in a real clinical setting (*n* = 84).

Variable	Frequency (N)	Percent (%)
**How comfortable you are with interviewing and managing a patient with a mental health problem in a real clinical setting?** - Extremely comfortable - Very much comfortable - Slightly comfortable - Not at all comfortable	23 13 33 15	27.4 15.5 39.3 17.8

**Table 3:** Recommendation of using a simulated patient as teaching method in teaching psychiatric mental health nursing (*n* = 84).

Variable	Frequency (N)	Percent (%)
**How much would you recommend using a simulated patient as a teaching method in teaching psychiatric and mental health nursing after your experience?** - Strongly recommended - Very much recommended - Average recommended - Strongly not recommended	47 12 18 7	56.0 14.3 21.4 8.3

**Table 4:** Frequency distribution of students’ satisfaction with learning scale items (*n* = 84).

Satisfaction Statement	1 Strongly Disagree		2 Disagree		3 Undecided		4 Agree		5 Strongly Agree		Mean (SD)
	N	%	N	%	N	%	N	%	N	%	
The teaching methods used in this simulation were helpful and effective.	9	10.1	7	8.3	11	13.1	16	19.0	41	48.8	3.87 (1.39)
2. The simulation provided me with a variety of learning materials and activities to promote my learning the psychiatric and mental health-nursing curriculum.	10	11.9	5	6	12	14.3	18	21.4	39	46.4	3.85 (1.38)
3. I enjoyed how my instructor taught the simulation.	8	9.5	6	7.2	11	13.1	20	23.8	39	46.4	3.90 (1.32)
4. The teaching materials used in this simulation were motivating and helped me to learn.	9	10.7	10	11.9	10	11.9	14	16.7	41	48.8	3.81 (1.43)
5. The way my instructor(s) conducted the simulation sessions was suitable to the way I learn.	7	8.3	7	8.3	11	13.1	19	22.6	40	47.6	3.93 (1.31)

Total satisfaction with learning via simulation scale score: (M ± SD) 19.36 ± 6.32.

**Table 5:** Frequency distribution of students’ level of self-confidence in learning (*n* = 84).

Self-Confidence Statement	1 Strongly Disagree		2 Disagree		3 Undecided		4 Agree		5 Strongly Agree		Mean (SD)
	N	%	N	%	N	%	N	%	N	%	
I am confident that I am mastering the content of the simulation activities that my instructors presented to me.	8	9.5	8	9.5	8	9.5	24	28.6	36	42.9	3.86 (1.33)
2. I am confident that this simulation covered critical content necessary for the mastery of psychiatric and mental health nursing curriculum.	8	9.5	6	7.1	9	10.7	26	30.9	35	41.7	3.88 (1.29)
3. I am confident that I am developing the skills and obtaining the required knowledge from this simulation to perform necessary tasks in a clinical setting.	9	10.7	8	9.5	10	11.9	23	27.4	34	40.5	3.77 (1.36)
4. My instructors used helpful resources to conduct the simulation.	5	6	6	7.1	11	13.1	25	29.8	37	44.0	3.99 (1.19)
5. It is my responsibility as the student to learn what I need to know from this simulation activity.	8	9.5	6	7.1	11	13.1	22	26.2	37	44.0	3.88 (1.31)
6. I know how to get help when I do not understand the concepts covered in the simulation sessions.	6	7.1	4	4.8	13	15.5	22	26.2	39	46.4	4 (1.21)
7. I know how to use simulation activities to learn critical aspects of the psychiatric and mental health nursing skills.	9	10.7	3	3.6	13	15.5	26	31	33	39.2	3.85 (1.28)
8. It is the instructor’s responsibility to tell me what I need to learn of the simulation activity content during class time.	11	13.1	5	6	20	23.8	15	17.9	33	39.2	3.64 (1.39)

Total self-confidence with simulation learning scale score: (M ± SD): 30.87 ± 9.1.

**Table 6:** Relationship between satisfaction with learning and past experience with simulation, simulation training, and past experience with a person with a mental health problem among study participants (*n* = 84).

Variable	Mean	F	*p*
**Do you have any past experience with simulation?** Yes No	19.83 17.85	1.500	0.224
**Have you ever received any simulation training?** Yes No	19.93 18.28	1.300	0.258
**Do you have any past experience with a person experiencing mental health problem?** Yes No	18.32 20.68	2.943	0.090

**Table 7:** Relationship between Students’ Self-confidence in Learning Scale score and past experience with simulation, simulation training, and past experience with a person with a mental health problem among study participants (*n* = 84).

Variable	Mean	F	*p*
**Do you have any past experience with simulation?** Yes No	31.27 29.60	0.508	0.478
**Have you ever received any simulation training?** Yes No	31.38 29.90	0.503	0.480
**Do you have any past experience with a person experiencing mental health problem?** Yes No	29.53 32.57	2.342	0.129

**Table 8:** Relationship between total SSLS scores and past experience with simulation, simulation training, and past experience with a person with a mental health problem among study participants *(n* = 84).

Variable	Mean	F	*p*
**Do you have any past experience with simulation?** Yes No	51.09 47.45	0.882	0.350
**Have you ever received any simulation training?** Yes No	51.31 48.17	0.814	0.369
**Do you have any past experience with a person experiencing mental health problem?** Yes No	47.85 53.24	2.682	0.105

**Table 9:** Correlation between satisfaction, self-confidence, total SSLS and students’ GPA (*n* = 84).

Variable	Students’ GPA	
	**r**	***p***
**Satisfaction with learning**	0.127	0.250
**Self-confidence in learning**	0.107	0.329
**Total SSLS**	0.118	0.286

**Table 10:** Correlation between satisfaction, self-confidence, total SSLS, and students’ recommendation to use simulation in psychiatric and mental health education (*n* = 84).

Variable	Students’ Recommendation of Using Simulation in Psychiatric Nursing Education	
	**F**	***p***
**Satisfaction with learning**	4.96	0.003 *
**Self-confidence in learning**	2.56	0.06
**Total SSLS**	3.54	0.01 *

* Significant.

## Data Availability

The raw data supporting the conclusions of this article will be made available by the corresponding author on request.

## Public Involvement Statement

No public involvement in any aspect of this research.

## Guidelines and Standards Statement

This study was designed following the statement ‘Strengthening the Reporting of Observational Studies in Epidemiology (STROBE) for observational studies.
